# Intra-annual raw basal area increments (early-wood and late-wood) of *Pinus nigra* subsp. *laricio* Poiret trees from southern Italy at the pines׳ mesic to xeric distribution range

**DOI:** 10.1016/j.dib.2018.08.095

**Published:** 2018-08-29

**Authors:** Gianluigi Mazza, Dimitrios Sarris, Ugo Chiavetta, Rossana M. Ferrara, Gianfranco Rana

**Affiliations:** a CREA Research Centre for Forestry and Wood, 52100 Arezzo, Italy; b Faculty of Pure and Applied Sciences, Open University of Cyprus, Latsia, 2252 Nicosia, Cyprus; c Department of Biological Sciences, University of Cyprus, 1678 Nicosia, Cyprus; d School of Environmental Studies, KES College, 1055 Nicosia, Cyprus; e CREA Research Centre for Agriculture and Environment, 70125 Bari, Italy

## Abstract

This article contains tree rings data related to the research article entitled “An intra-stand approach to identify intra-annual growth responses to climate in *Pinus nigra* subsp. *laricio* Poiret trees from southern Italy” (Mazza et al., 2018). Most dendroclimatological studies on black pine have been conducted on the *P. nigra* subsp. *nigra,* while only few results on climate-growth relationships are available for other taxa such as *P. nigra subsp. laricio,* which has the narrowest distribution range of the collective species *P. nigra*. This data article provides tree rings data for the subsp. *laricio* at an intra-annual growth level, distinguishing early-wood (EW) and late-wood (LW), from an even aged forest stand from the Sila mountain area within the subspecies mesic to xeric distribution range.

**Specifications Table**

**Table:** 

Subject area	Ecology
More specific subject area	Dendrochronology, Dendroecology
Type of data	Excel file of EW and LW basal area increments
How data was acquired	Tree cores were extracted using a 5-mm-diameter increment borer from dominant straight trees
Data format	Raw data in.xls
Experimental factors	–
Experimental features	–
Data source location	Bonis basin, Sila Greca - 39° 28′ N; 16° 32′ E, Calabria (Italy)
Data accessibility	With this article
Related research article	Mazza, G., Sarris, D., Chiavetta, U., Ferrara, R.M., Rana, G., 2018. An intra-stand approach to identify intra-annual growth responses to climate in *Pinus nigra* subsp. *laricio* Poiret trees from southern Italy. For. Ecol. Manage., 425: 9-20.

**Value of the data**

• This dataset provides early-wood and late-wood basal area increments of *Pinus nigra* subsp. *laricio,* which has the narrowest distribution range of the collective species *P. nigra*.
• The intra-annual growth analysis can reveal an in-depth tree growth variability and trends compared to the tree-ring widths measured at the typical annual resolution.
• The distinction between early-wood and late-wood provides a detailed analysis of the climate influence within the growing season as it can reveal the main climate-driven patterns of intra-annual growth variations.

## 1 Data

Earlywood (EW) and latewood (LW) widths were measured from bark to pith with a 0.01-mm precision by a computer-linked mechanical platform (LINTAB 6) under a stereoscope and a software package (Time Series Analysis and Presentation, TSAP, Frank Rinn, Heidelberg, Germany). The EW-LW transition was visually identified by a detectable changing in cell wall thickness, resulting in a band of darker cells.

## 2 Experimental design, materials, and methods

### 2.1 Dendrochronological analysis

Dominant straight trees, within a strict diameter range to avoid a potential heterogeneous effect of tree size on growth responses to climate, were cored at breast height using a 5-mm-diameter increment borer. One or two cores per tree were extracted, according to the symmetric shape of each stem. Before measuring, the increment cores were prepared according to the standard dendrochronological procedures. See [1] for detailed information. Each ring width series was first visually checked and then statistically verified for cross-dating and measurement errors using the dendrochronology program library in R “dplR” [2].

The basal area increment (BAI) method was employed as it provides a better proxy for the three dimensional mass increment than ring width. It avoids the effect of reduction in ring widths due to the diameter increase of trees (i.e. it is free of age trend) without eliminating the patterns of increase or decrease in ring width due to other causes. BAI, in cm², was derived using the following equation implemented in the R package “dplR”: BAIt = π(*wt*² + 2*wtR*(*t*−1)) where wt is the annual ring width and R(*t*−1) is the stem radius at the beginning of the annual increment.
